# Steroids and Related Compounds: Basic and Clinical Aspects 

**DOI:** 10.1155/2013/703476

**Published:** 2013-02-03

**Authors:** Fátima Regina Mena Barreto Silva, Leila Zanatta, Rozangela Curi Pedrosa, Ming-Zhu Fang

**Affiliations:** ^1^Departamento de Bioquímica, Universidade Federal de Santa Catarina, Caixa Postal 5069, 88040-970 Florianópolis, SC, Brazil; ^2^Health Sciences Area, Universidade Comunitária da Região de Chapecó, Avenida Senador Attílio Fontana 591E, 89809-000 Chapecó, SC, Brazil; ^3^Department of Environmental and Occupational Medicine, Robert Wood Johnson Medical School, University of Medicine & Dentistry of New Jersey, 170 Frelinghuysen Road, Piscataway, NJ 08854, USA

The steroid hormones generate myriad effects through several well-known mechanisms of action. Beyond the physiological function of steroid hormones, the steroid-like effects of some natural and synthetic compounds have led to the promising field of alternative therapies. However, to these new compounds, the molecular, subcellular, and cellular signal transductions need to be elucidated. Although new questions about the relevance of multiple targets of action for exogenous compounds are deeply in discussion, the original contributions that depict novel insights into basic and clinical aspects with perspectives on medical application are welcome.

This special issue compiles papers from renowned research groups in the world that cover the frontiers of the latest findings on steroids and related compounds effect, mechanism of action and its relevance on carcinogenesis, immune suppression, and osteoporosis, as well as novel steroidal glycosides characterization. 

The papers on sex steroids hormones review the role of progesterone (precursor for androgens and estrogens produced by the gonadal and adrenal cortical tissues) and related progestins compounds in hepatocellular carcinoma. The authors highlight that the higher incidence of hepatocellular carcinoma in men than women might have resulted from the stimulatory effects of androgen and the protective effects of estrogen and also eventually suggest a new insight into the associations of progesterone and related compounds with hepatocellular carcinoma development and treatment. Also, the sex steroid hormones in the modulation of bacterial-host interactions were revised since the dimorphic sex difference (low immune responses presented in males as compared to females) is mainly due to the differential modulation of the immune system by sex steroid hormones through the control of proinflammatory and anti-inflammatory cytokines expression, as well as Toll-like receptors expression and antibody production.

Some interesting studiesonhumans addressed the effect of chronic glucocorticoid therapy on osteoporosis in children with 21-hydroxylase deficiency as much to replace congenital deficits in cortisol synthesis as to reduce androgen secretion by adrenal cortex. As consequence, a secondary osteoporosis is formed. It results in an early, transient increase in bone resorption accompanied by decrease in bone formation, maintained for the duration of glucocorticoid therapy. Based on conflicting results from the literature about the bone status on glucocorticoid-treated patients with 21-hydroxylase deficiency, the authors point that the monitoring of the bone status of these patients, checking bone mineral density and bone turnover markers, and studying the expression of regulators of bone resorption should be useful in order to avoid glucocorticoid-induced osteoporosis in adulthood. Also, based on many epidemiological studies concerning the inverse relationship between isoflavone intake and bone loss and fracture rate, some substances on serum levels after food intake indicated for patients with osteopenia/osteoporosis were analyzed. Concerning genistein bioavailability, it was deeply discussed that, beyond the intestinal bacteria, solubility and permeability, glucosidase activity, viscosity induced by food additives, and a multitude of transporters on luminal intestinal cells for absorption, several factors from the diet composition influence the net absorption of single entity and also the effectiveness of the bone build. So, with these data in mind, the bioavailability of genistein depends on specific ingredients and excipients in each formulation which can interfere with absorption and could have clinical implications on efficacy. 

The ongoing investigations of some groups in the world have characterized new steroidal compounds from plants with medicinal interest. Three new steroidal glycosides, named as stauntosides L, M, and N, along with one known C_21_ steroidal glycoside, anhydrohirundigenin monothevetoside, were isolated from the roots of *Cynanchum stauntonii* (Decne.) and extensively evaluated by spectroscopic analyses, mainly 1D and 2D NMR, HRESI-MS and chemical methods. It is known that C_21_ steroids and their glycosides are of considerable bioactivities, such as hypolipidemic and antitumor activity. So the enriched information about *C. stauntonii* as a significant source of steroidal glycosides deserves careful phytochemistry investigation as well as the classification of bioactive compounds to be proposed as nutraceutical agents interesting both to academy and industry and also to specific therapy option.

